# High-Crystallinity BiOCl Nanosheets as Efficient Photocatalysts for Norfloxacin Antibiotic Degradation

**DOI:** 10.3390/nano13121841

**Published:** 2023-06-12

**Authors:** Dongxue Song, Mingxia Li, Lijun Liao, Liping Guo, Haixia Liu, Bo Wang, Zhenzi Li

**Affiliations:** 1 Key Laboratory of Functional Inorganic Material Chemistry, Ministry of Education of the People’s Republic of China, School of Chemistry and Materials Science, Heilongjiang University, Harbin 150080, China; 2 Shandong Provincial Key Laboratory of Molecular Engineering, School of Chemistry and Chemical Engineering, Qilu University of Technology (Shandong Academy of Sciences), Jinan 250353, China

**Keywords:** photocatalysis, BiOCl, nanosheets, high-crystallinity

## Abstract

Semiconductor photocatalysts are essential materials in the field of environmental remediation. Various photocatalysts have been developed to solve the contamination problem of norfloxacin in water pollution. Among them, a crucial ternary photocatalyst, BiOCl, has attracted extensive attention due to its unique layered structure. In this work, high-crystallinity BiOCl nanosheets were prepared using a one-step hydrothermal method. The obtained BiOCl nanosheets showed good photocatalytic degradation performance, and the degradation rate of highly toxic norfloxacin using BiOCl reached 84% within 180 min. The internal structure and surface chemical state of BiOCl were analyzed using scanning electron microscopy (SEM), transmission electron microscopy (TEM), Raman, Fourier transform infrared spectroscopy (FTIR), UV–visible diffuse reflectance (UV-vis), Brunauer–Emmett–Teller (BET), X-ray photoelectron spectra (XPS), and photoelectric techniques. The higher crystallinity of BiOCl closely aligned molecules with each other, which improved the separation efficiency of photogenerated charges and showed high degradation efficiency for norfloxacin antibiotics. Furthermore, the obtained BiOCl nanosheets possess decent photocatalytic stability and recyclability.

## 1. Introduction

With the rapid development of global industry, environmental pollution is becoming increasingly severe. Global environmental pollution and ecological damage have released many toxic and harmful pollutants in water sources and soil [1,2,3,4,5,6,7]. In recent years, norfloxacin has been detected in different water resources and drinking water. Norfloxacin antibiotic tends to have an aromatic structure, making it difficult to degrade. This antibiotic can damage ecosystems and health due to its high polarity and low volatility. If this antibiotic is introduced into the body through drinking water, severe drug resistance will be caused. Given the ongoing contamination of the environment by norfloxacin, researchers are eager to develop an efficient and practical strategy to remove norfloxacin antibiotics from the water environment. Photocatalysis is a promising green technology for water restoration and sustainable development. This technology can be the safest and most effective method in environmental remediation due to the utilization of the unique semiconductor properties of catalysts and natural sunlight to remove pollutants from water [8,9,10,11,12,13].

Since photocatalysis was first reported in the 1970s, titanium dioxide was the first photocatalyst used in the field of photocatalysis due to its advantages of being green and low price. However, its low quantum efficiency and poor visible light absorption have led to the development of new photocatalytic materials. Thus, BiOCl has come into the limelight and developed in various fields, such as environment and energy, because of its typical laminar structure. Nevertheless, the catalytic activity of BiOCl is tremendously restricted by its low photoconversion rate and high electron–hole recombination rate. In order to resolve the shortcomings of BiOCl materials, researchers have performed various modifications on BiOCl, including defect formation, heteroatom doping, metal deposition, and construction of heterojunction.

Bismuth chloride oxide (BiOCl) is gradually being viewed as a critical ternary photocatalyst with a layered structure of alternating [Bi_2_O_2_]^2+^ layers and double Cl^−^ atomic layers along the *c*-axis [14,15,16]. The layered structure can hinder in electron–hole recombination due to the weak van der Waals force interactions between the [Bi_2_O_2_]^2+^ layer and the double Cl^−^ atomic layer [17,18,19]. This structure can also produce uneven charge distribution between the cation and anion layers, the polarization of the constituent atoms and orbitals, internal electric field, and the surface capture of photogenerated carriers [20,21,22,23,24]. The atomic and orbital polarized intra-layer structure has enough polarized orbital space, which directly promotes the electron–hole separation rate and enhances the activity of the photocatalyst [25]. Liao et al. [26] synthesized BiOCl nanowires using a simple and safe method to change the conventional two-dimensional structure of BiOCl into a one-dimensional structure with a high spreading ratio and exposing many surface active sites. This structure leads to the curing of the wide bandgap of BiOCl, thereby creating a narrow bandgap semiconductor with a bandgap position of about 1.92 eV. The photocatalysts present enhanced visible light absorption ability and rhodamine B dye degradation activity with up to 80% removal efficiency in 90 min. Furthermore, Zhang et al. [27] assembled three-dimensional nanoflowers with nanosheets of highly exposed (001) facets. Its UV absorption spectrum exhibited an absorption edge at 358 nm, and the estimated bandgap gap was 3.46 eV. The photocatalytic removal efficiency of rhodamine B using BiOCl nanoflowers was up to 98% within 60 min, which was 34% higher than that of BiOCl nanoflakes. Due to the wide bandgap, BiOCl photocatalysts exhibit valuable photocatalytic activity in the UV range. However, their photocatalytic characteristics are rarely represented individually in various studies.

Herein, high-crystallinity BiOCl was synthesized using the conventional hydrothermal method with Bi(NO_3_)_3_·5H_2_O as the Bi source to form a large number of 1~2 μm nanosheets. The high crystallinity suggests that most molecular chains are tightly ordered and compact in the microscopic state, thus leading to enhanced intermolecular forces. In macroscopic phenomena, it shows high catalyst stability. The chemical surface states and molecular internal motion trajectories of BiOCl based on its high crystallinity were thoroughly studied.

## 2. Experimental Section

### 2.1. Chemicals

Bismuth nitrate pentahydrate (Bi(NO_3_)_3_·5H_2_O) was purchased from Shanghai Dibai Biotechnology Co., Ltd. (Shanghai, China), and potassium chloride (KCl) was obtained from Tianjin Guangfu Technology Development Co., Ltd. (Tianjin, China) Bi(NO_3_)_3_·5H_2_O and KCl are of analytical grade and used without further purification.

### 2.2. Synthesis

6 mmol Bi(NO_3_)_3_·5H_2_O and 6 mmol KCl were first added to 32 mL distilled water. Then, the solution was stirred for 30 min. The above solution was subsequently transferred to a 50 mL PTFE-lined stainless steel autoclave for continuous reaction at 160 °C for 12 h (24 h). The collected sample was denoted as BiOCl (BiOCl 24 h).

### 2.3. Characterizations

Powder X-ray diffraction (XRD) patterns were analyzed using Bruker D8 Advance diffractometer with monochromatic Cu Kα Radiation (λ = 1.5406, acceleration voltage 40 kV, applied current 20 mA). Raman spectra were analyzed using Jobin Yvon HR 800 micro Raman spectrometer at 457.9 nm. The refined structure and morphology of the samples were recorded on scanning electron microscopy (SEM, FEI Sirion 200 instrument operated at 15 kV) and transmission electron microscopy (TEM, Tecnai G2 F20, acceleration voltage 200 kV). The surface state of samples were analyzed using X-ray photoelectron spectroscopy (XPS), which consists of a monochromatic Al Ka (1253.6 eV) as the X-ray source. The optical properties of the samples were analyzed by UV–vis diffuse reflectance spectroscopy (DRS) using a λ950 UV–vis spectrophotometer in which BaSO_4_ was used as the reference. The nitrogen adsorption–desorption isotherms at 77 K were collected using the AUTOSORB-1 (Quantachrome instrument Co., Ltd. (Beijing, China)) nitrogen adsorption instrument. The Bruner–Emmett–Taylor equation is used to estimate the specific surface area. The pore size distribution was measured using Barrett–Joyner–Halenda (BJH) measurements from the isotherm adsorption branch. Fourier transform infrared spectroscopy (FT-IR) was recorded with Perkin–Elmer Spectrum One spectrometer using KBr particles.

### 2.4. Photocatalytic Performance Test

The photocatalytic activity of BiOCl was tested in the norfloxacin (C_16_H_18_FN_3_O_3_) degradation process. A 300 W Xe lamp (Beijing Porphyry Co., Ltd. (Beijing, China)) with an AM 1.5 filter was used as the light source. The calibrated light intensity was 100 MW cm^−2^ before the measurements. Typically, the prepared photocatalyst (50 mg) was first added to 50 mL of norfloxacin solution (10 mg/L). The suspension was placed in the dark at room temperature for 60 min to achieve the adsorption–desorption equilibrium. Then, the suspension was irradiated and vigorously stirred, and 4 mL of the reaction mixture was taken every 30 min and filtered through a 0.22 μm microporous filter to remove the photocatalyst from the solution. The UV–Vis absorption spectra of the residual norfloxacin were recorded with a UV-1800 BPC UV–Vis spectrophotometer (Shanghai Mepro Delta Co., Ltd. (Shanghai, China)).

### 2.5. Photoelectrochemical Measurements

With Ag/AgCl as reference electrode, platinum plate as counter electrode, FTO glass sprayed with BiOCl or BiOCl 24 h material as photoanode, and 0.2 M Na_2_SO_4_ aqueous solution as electrolyte (the solution was degassed with nitrogen for one hour before use), the photoelectrochemical properties were analyzed using an electrochemical workstation (Princeton Versa STAT) in the three-electrode system. The electrochemical test conditions are as follows: the AC characteristic was set as the starting frequency of 10,000 Hz, the ending frequency of 0.1 Hz, DC potential vs. OC potential, and the overvoltage was 600 mV. The preparation of the photoanode was carried out using a traditional spraying method. A 50 mg sample was dispersed in 5 mL ethanol solution and sonicated for more than 1 h to achieve uniform dispersion. Then, the uniformly dispersed solution was sprayed on the transparent FTO glass substrate (TCO, fluorine-doped tin oxide layer, Nippon flat glass in Japan) with a spray gun. The sprayed FTO glass was calcined at 350 °C for 2 h at a constant heating rate of 5 °C/min in a N_2_ atmosphere. In the photoelectrochemical test, the coating area and illumination area are 1.5 cm^2^, and A 300 W Xe lamp (Beijing Porphyry Co., Ltd. (Beijing, China)) with an AM 1.5 filter was used as the light source.

## 3. Results and Discussion

### 3.1. Morphology and Microstructure

**Figure 1:**
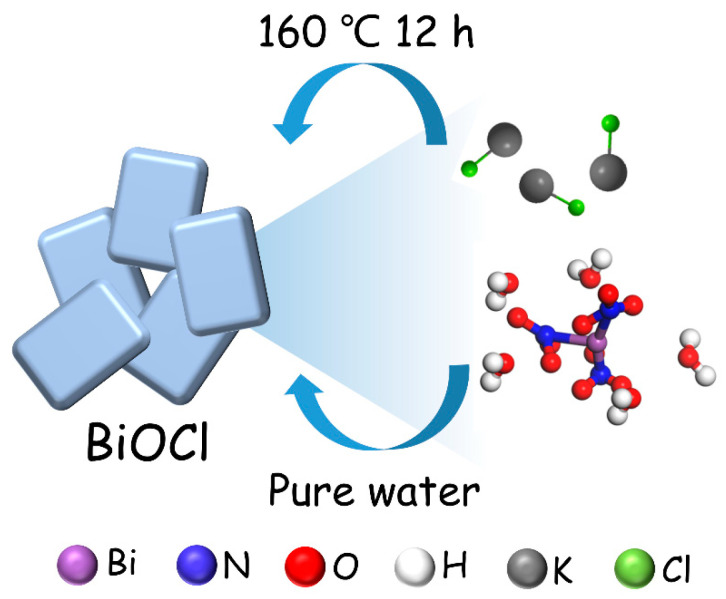
Scheme for the formation of BiOCl.

The synthesis process of creating highly crystalline BiOCl nanosheets using a one-step hydrothermal method is described in Figure 1. As described in Figure 1, Bi(NO_3_)_3_·5H_2_O is used as the Bi source, while KCl provides the Cl. The BiOCl nanosheet structure can be obtained using continuous heating at 160 °C in a pure water solvent for 12 h.

**Figure 2:**
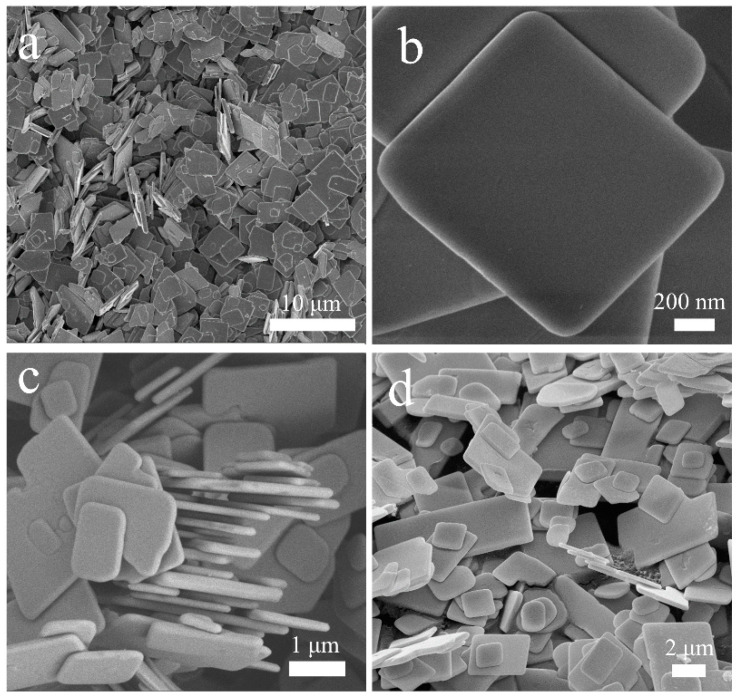
SEM images of BiOCl (**a**–**c**) and BiOCl 24 h (**d**).

Figure 2a–c show scanning electron microscopy (SEM) images of BiOCl, showing that the BiOCl structure is a two-dimensional (2D) nanosheet with uniform morphology. The width of the nanosheet ranges from 1 to 2 μm, and the thickness ranges from 0.4 to 0.6 μm. The formed nanosheets are clustered together by electrostatic interaction because the BiOCl crystal layers are tightly bound by chemical bonds and the layers are connected by weak van der Waals forces between the layers, and the unique layered structure leads to the easy formation of BiOCl nanosheets with high aspect ratios [28,29,30,31]. In the magnified image, it can be clearly observed that the material’s surface is smooth and free from particles, thus increasing the material’s wear resistance. The SEM image of BiOCl 24 h is shown in Figure 2d. Although it has a similar nanosheet structure, its morphology is irregular and has tiny impurities.

**Figure 3:**
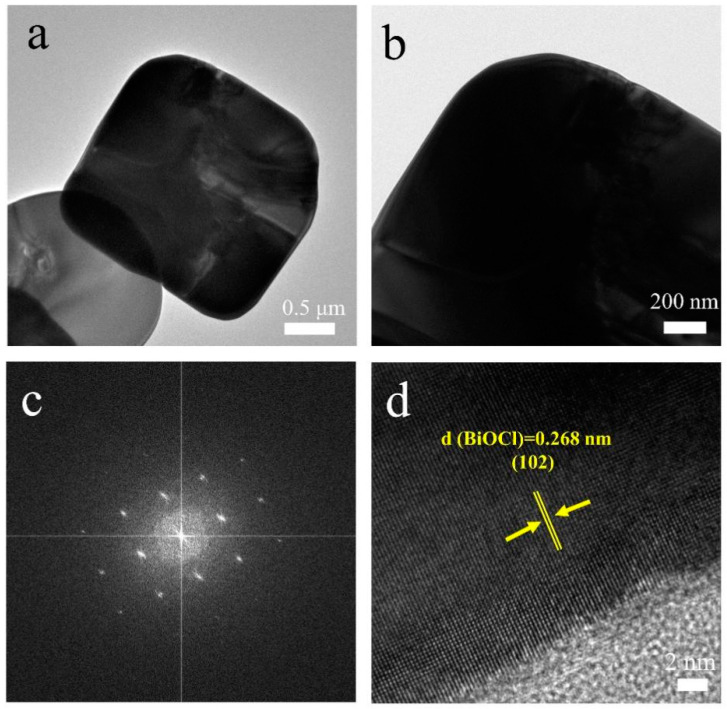
TEM (**a**,**b**), SAED (**c**), and HRTEM (**d**) images of BiOCl.

The transmission electron microscopy (TEM) images of Figure 3a,b present the nanosheet structure of single-phase BiOCl and the appearance of regular and clear arrays of square diffraction spots in selected areas of electron diffraction (SAED) images of the corresponding nanosheets (Figure 3c), indicating that BiOCl is a single-crystal material [32]. The high-resolution transmission electron microscopy (HRTEM) in Figure 3d demonstrates clear lattice stripes. The exposed (102) crystal plane of BiOCl has a lattice spacing of 0.268 nm.

**Figure 4:**
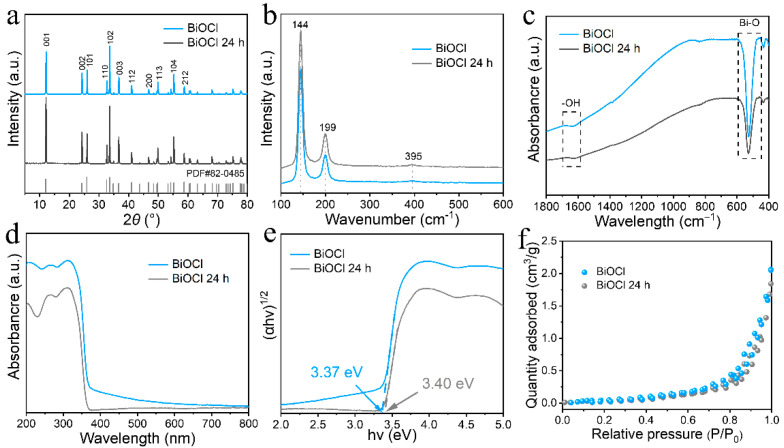
XRD patterns (**a**), Raman spectra (**b**), FT-IR (**c**), UV–Vis diffuse absorption spectra (**d**), bandgap (**e**), and N_2_ adsorption–desorption isotherms (**f**) of BiOCl and BiOCl 24 h, respectively.

The XRD pattern in Figure 4a analyzes the crystal structures of BiOCl and BiOCl 24 h. All the diffraction peaks correspond well to the BiOCl tetragonal structure crystalline phase, which has very high crystallinity and no other spurious peaks, indicating that there is only one substance, i.e., BiOCl, in the material. Its corresponding standard card is JCPDS no. 82-0485, and the space group is P4/mmm (NO. 129). The cell parameters of BiOCl are a = b = 3.878 Å and c = 7.403 Å. The highly crystalline BiOCl and BiOCl 24 h have eleven major diffraction peaks in the XRD patterns, 2*θ* = 12.0, 24.2, 25.9, 32.5, 33.5, 36.6, 41.0, 46.7, 49.8, 55.2, and 58.7°, and the diffraction peaks are located on the crystal planes of (001), (002), (101), (110), (102), (003), (112), (200), (113), (104), and (212), respectively. High crystallinity also indicates excellent dimensional stability and tight molecular alignment within the structure that is less prone to fracture. To further investigate the surface state of the samples, Raman tests were performed on the BiOCl catalysts to determine the stretching or vibrational modes of the molecules. Three prominent diffraction peaks appear in the Raman (Raman) spectrum of Figure 4b, among which 144 and 199 cm^−1^ bands belong to the Bi-X bond stretching vibrations within *A*_1 g_ and *E*_g_ of BiOCl, respectively. The peak at 395 cm^−1^ can be ascribed to the *E*_g_ and *B*_1 g_ energy bands generated by the motion of O atoms [33,34]. It can be seen that the Raman vibrational peak of BiOCl is reduced compared to BiOCl 24 h, indicating that the intra-bond stretching of BiOCl is weakened and the absorption of light is stronger than BiOCl 24 h. To get a clear understanding of the structural atomic bonding within BiOCl, Fourier transform infrared spectroscopy (FTIR) was utilized to detect the chemical bonds in BiOCl molecules. As shown in Figure 4c, the vibrational peak at 1621 cm^−1^ can be attributed to the stretching and bending motion of the hydroxyl (–OH) group, which is caused by the surface hydration phenomenon. The strong characteristic peak at 526 cm^−1^ is generated by stretching of the Bi-O bond within BiOCl and BiOCl 24 h [35]. The formation of highly crystalline BiOCl was further verified. The optical properties were measured using UV–visible diffuse reflectance (UV-vis), as shown in Figure 4d. BiOCl has a continuous absorption band of 360–800 nm. This absorption band is better than the light absorption of BiOCl 24 h, indicating that BiOCl is more sensitive to light than BiOCl 24 h. It is determined that the maximum absorption wave edge occurs at about 378 nm. Thus, the bandgap (E_g_) position can be obtained by Equation (1):

E_g_ (eV) = 1240/λ (1)

The λ is the maximum absorption wavelength. The estimated bandgap value of BiOCl and BiOCl 24 h are about 3.37 and 3.40 eV, respectively (Figure 4e), and are suitable for light absorption in the UV region. Although UV light accounts for a relatively small fraction of solar energy, such a forbidden bandwidth, it can absorb UV light sufficiently to achieve the catalytic application of BiOCl in the UV region. As shown in Figure 4f, the nitrogen adsorption–desorption isotherm was obtained for the analysis of the porous structure. BiOCl and BiOCl 24 h in the relative high-pressure region exhibit type IV isotherm and H hysteresis loop. Type IV isotherm is similar to type II isotherm in the relative low-pressure region. However, it is prone to capillary coalescence in the region of higher relative pressure, making the latter half of the curve climb sharply [36]. It also indicates that BiOCl nanosheets are mesoporous structures. The specific surface area, pore volume, and pore diameter of BiOCl catalysts are further derived from the Brunauer–Emmett–Teller (BET) theory to give a total surface area of 1.0 m^2^g^−1^. The pore volume and pore diameter of BiOCl is 0.002 cm^3^g^−1^ and 16.6 nm, respectively.

**Figure 5:**
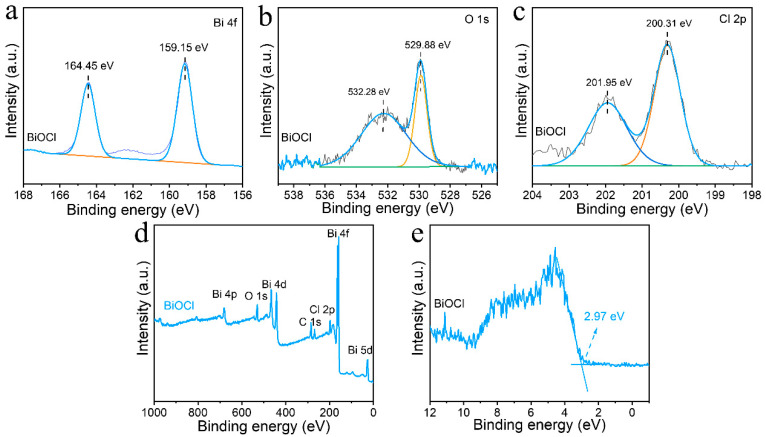
XPS spectra of Bi 4f (**a**), O 1s (**b**), Cl 2p (**c**), Full-scale XPS spectra (**d**), and valence band spectra (**e**) of BiOCl.

In addition to the internal structural analysis of BiOCl, we further investigated the surface elements of BiOCl and its chemical state. The X-ray photoelectron spectra (XPS) in Figure 5 perfectly demonstrate the elemental states in BiOCl. Among them, the fitted peaks of Bi^3+^ fall in the binding energy of 164.45 and 159.15 eV (Figure 5a), and the two peaks belong to Bi 4f_7/2_ and Bi 4f_5/2_ orbitals, respectively [37]. The fitted peaks of O elements are at 532.28 and 529.88 eV binding energy (Figure 5b), which can be ascribed to oxygen-containing components adsorbed to the surface of BiOCl and the hydroxyl and lattice oxygen in BiOCl. [38]. The two fitted peaks for elemental Cl at 201.95 and 200.31 eV (Figure 5c) correspond to the Cl 2p_3/2_ and Cl 2p_1/2_ orbitals, respectively, confirming the presence of Cl^−^ [39]. The elemental composition was also analyzed in the XPS full spectrum (Figure 5d), containing characteristic peaks of orbitals of the three elements, i.e., Bi, O, and Cl. Bi 4p, O 1s, Bi 4d, C 1s, Cl 2p, Bi 4f, and Bi 5d orbitals can be observed in the full spectrum. C 1s is from the carbon in air adsorbed on the sample’s surface during testing. For Bi elements, Bi 4f has the highest intensity, the smallest peak width, and the best symmetry and is the mainline spectrum of Bi elements. There are also weak line spectra of Bi 4p, Bi 4d, and Bi 5d. Since Bi elements have multiple internal electrons, multiple XPS signals of Bi elements were produced. In addition, there is no interference from any other elements in BiOCl. It indicates that the sample synthesized using BiOCl is clean and free of impurities. The XPS valence band spectra of BiOCl also provided the valence band structure information of BiOCl. The energy corresponding to the valence band position is around 2.97 eV as obtained in Figure 5e.

**Figure 6:**
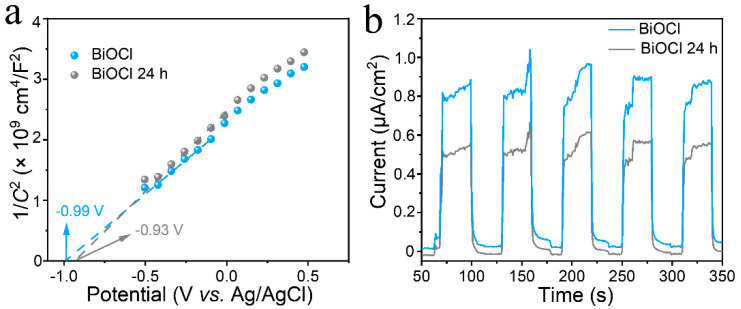
Mott–Schottky plot (**a**) and I-t (**b**) of BiOCl and BiOCl 24 h, respectively.

As shown in Figure 6a, the Mott–Schottky curves were tested to determine the flat-band valence. The intercept of the curves in the *x*-axis give the flat-band potentials of −0.99 and −0.93 V for BiOCl and BiOCl 24 h. It can be seen that BiOCl and BiOCl 24 h have positive slope curves, which proves that this material is an n-type semiconductor, and the participation in the conductivity is dominated by free electrons. The carrier concentration can be obtained from Equation (2):

(2) $$N_{d}=\left(\frac{2}{e_{0}\varepsilon \varepsilon_{0}}\right){\left(\frac{d\frac{1}{C^{2}}}{dV}\right)}^{-1}$$

where *e*_0_ is the charge per unit charge, *ε* is the relative permittivity, *ε*_0_ is the vacuum permittivity, *C* is the interfacial capacitance, and *V* is potential. We obtain the vacuum dielectric constant of BiOCl is *ε*_0_ = 55 [40]. The carrier concentration of BiOCl and BiOCl 24 h were calculated as *N*_d_ = 1.22 × 10^19^ and *N*_d_ = 1.07 × 10^19^, respectively. Furthermore, the flat-band potential can be deduced from the position of the conduction band (CB) by using Equation (3):

*E* (RHE) = *E* (Ag/AgCl) + 0.0591 pH + 0.197 (3)

*E* is the electrode potential. The calculated CB value of BiOCl and BiOCl 24 h fall at −0.38 and −0.32 V, respectively. The instantaneous photocurrent responses (I-t) of BiOCl and BiOCl 24 h are shown in Figure 6b. The photocurrent of BiOCl is more significant than that of BiOCl 24 h, indicating that BiOCl has higher charge separation efficiency and a longer lifetime of photogenerated carriers. The enhancement of the transient photocurrent response is consistent with the results of the subsequent degradation performance. As a classical photocatalyst suitable for degradation, the oxidation capacity of BiOCl itself deserves to be studied in depth.

### 3.2. Photocatalytic Activity and Mechanism

**Figure 7:**
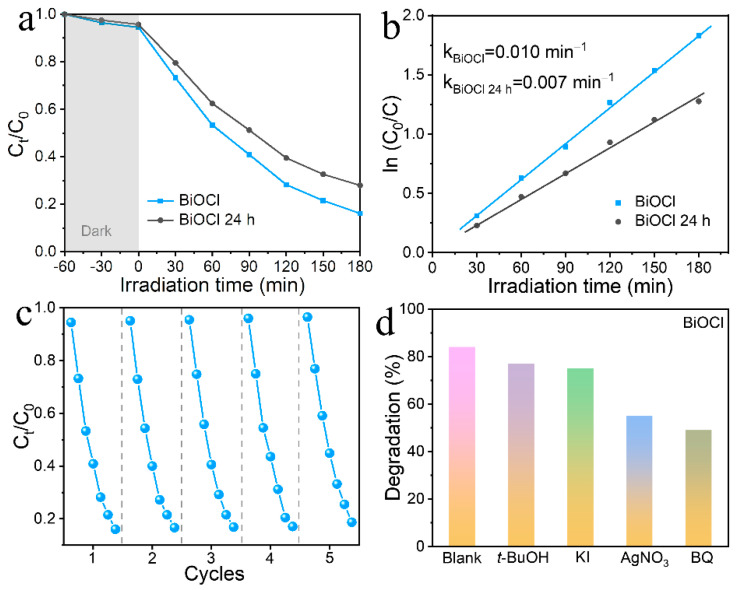
Photocatalytic degradation curves of norfloxacin (**a**). Rate curves (**b**) of BiOCl and BiOCl 24 h. Cyclic photodegradation curve for norfloxacin (**c**) of BiOCl. Inhibition of norfloxacin degradation by free radical scavengers in the BiOCl system (**d**).

Although the content of antibiotics in water resources is not high, it is still harmful to human beings and cannot be ignored. In this work, the degradation of norfloxacin in an aqueous solution was carried out under simulated sunlight irradiation using BiOCl as the photocatalyst. As seen in Figure 7a, after one hour of dark treatment to reach an adsorption balance of norfloxacin on BiOCl, the degradation rate of norfloxacin reached 84% within 180 min under light irradiation. In contrast, the degradation rate of norfloxacin over BiOCl 24 h was only 72%, indicating that BiOCl under 12 h reaction conditions possessed a better degradation ability. Moreover, the rate constants of 0.010 min^−1^ and 0.007 min^−1^ were obtained for BiOCl and BiOCl 24 h from the rate curves, respectively (Figure 7b). The rate constant of BiOCl was about 1.43 times higher than that of BiOCl 24 h, which once again highlighted that the BiOCl degradation process was efficient. Furthermore, some comparisons of the degradation performance of BiOCl photocatalysis are listed in Table 1, reflecting the good photocatalytic activity of BiOCl nanosheets evaluated in this work. To examine the recyclability of the BiOCl material, five cycling experiments were performed (Figure 7c). After five consecutive degradations, the catalytic activity remained almost unchanged. Such stability of BiOCl material indicates that it can be recycled after several uses in wastewater treatment. In the radical capture experiments in Figure 7d, tert-butanol (*t*-BuOH) was used to capture hydroxyl groups, potassium iodide (KI) to capture holes, silver nitrate (AgNO_3_) to capture electrons, and benzoquinone (BQ) to capture superoxide anions, respectively [41,42]. Compared with the blank experiment, we know that the main active substances in the BiOCl system are electrons and superoxide anions. In conclusion, BiOCl photocatalysts have both great photocatalytic activity and excellent recyclability, further confirming the feasibility of BiOCl in practical applications [43,44].

**Figure 8:**
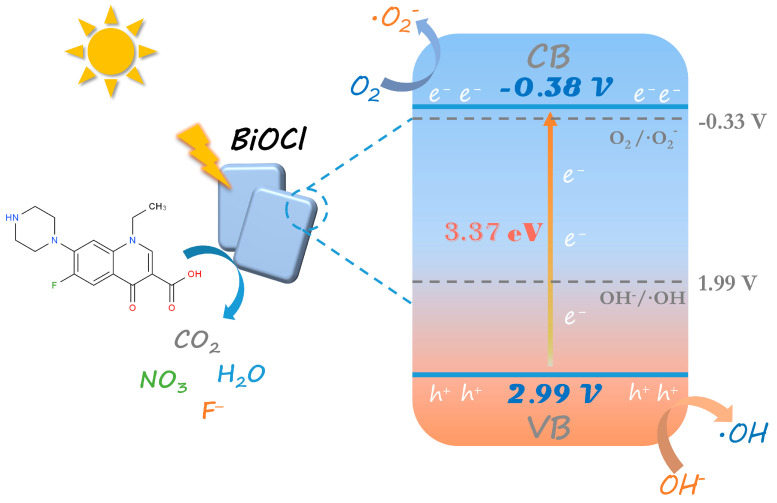
Photocatalytic mechanism of high-crystallinity BiOCl under sunlight irradiation.

As shown in the photocatalyst mechanism in Figure 8, the separation of electrons and holes occurs in highly crystalline BiOCl nanosheets under light irradiation. The excited electrons are transferred from the valence band to the conduction band, and the holes are left in the valence band. The CB value of BiOCl obtained from Equation (3) is −0.38 V, and the bandgap value obtained from Equation (1) is 3.37 eV. Therefore, the VB position can be obtained to be 2.99 V. The hydroxide (OH^−^) at the VB position loses an electron and is converted into a hydroxyl radical (^•^OH). In contrast, the oxygen (O_2_) molecule at the CB position accepts an electron and produces the reduction product, i.e., superoxide anion (^•^O_2_^−^). Both of them play an essential role in the photocatalytic reaction. Norfloxacin was photocatalytically converted to carbon dioxide, water, and other small molecular impurities over BiOCl materials. The photocatalytic carrier complex is prevented under photoexcitation, which promotes the oxidation ability of BiOCl, increases the active site, captures the norfloxacin molecule, and degrades norfloxacin into a non-toxic and harmless substance. Therefore, BiOCl with high crystallinity has excellent photocatalytic activity and exhibits significant wear and chemical resistance [54].

Researchers can adopt various modification methods to further enhance performance based on BiOCl nanosheets. First, defects created by detaching oxygen atoms in the lattice can trap electrons, thus preventing the recombination of electron–hole pairs and increasing the carrier concentration. This method can enhance the activity of semiconductor photocatalysts without introducing other impurities and is one of the most common methods for photocatalyst modification. Second, doping metal elements allows precise control of impurity distribution, high purity of doping, and excellent uniformity. Then, the energy band structure of the semiconductor can be adjusted to improve the light absorption and the transmission distance between the valence and conduction bands of electrons. Third, metal deposition can form a plasmon resonance effect or create Schottky junctions. Both of them enhance the transfer of photogenerated electrons in different electron migration modes, thus strengthening the absorption capacity of light and improving catalytic efficiency. Fourth, constructing heterojunctions with narrow bandgap semiconductors can expand the light absorption range, enhance the stability and prolong the carrier lifetime. Composites with narrow bandgap semiconductors not only modulate the performance of a single photocatalyst but also cause many new photochemical and photophysical properties.

## 4. Conclusions

In summary, high-crystallinity BiOCl nanosheets synthesized using a one-step hydrothermal method exhibited excellent photocatalytic activity. Under solar irradiation, BiOCl showed a 1.17-fold higher degradation rate and 1.43-fold higher rate constant than BiOCl 24 h in the degradation of highly toxic norfloxacin and exhibited excellent photocatalytic stability. The good degradation effect of BiOCl is due to the wide bandgap of BiOCl, which can absorb the UV region and generate photogenerated carriers. The oxidation ability of BiOCl is enhanced by the synergistic action of two photogenerated carriers, electrons, and holes. Furthermore, the high crystallinity of BiOCl microscopically shows strong intermolecular forces, which makes the BiOCl structure strong and stable. Macroscopically, it shows high tensile strength, i.e., the material is not easily damaged, and has good cyclic stability for pollutant degradation. Such high-crystallinity BiOCl materials provide a great basis for preparing composite photocatalysts and provide a strategy for building highly crystalline catalyst materials.

## Figures and Tables

**Table 1:** Comparison of the degradation performance of BiOCl photocatalysts in this work with other existing literature.

BiOCl Morphology	Light Source	Pollutant	Irradiation Time	Degradation Rate	Ref.
Nanosheets	300 W Xe lamp A.M 1.5	Norfloxacin	180 min	84%	This work
Nanosheets	10 W UV light 254 nm	Perfluorooctanoic acid	3 h	78%	[45]
Nanosheets	10 W UV light 254 nm	Perfluorooctanoic acid	24 h	59.3%	[46]
Nanosheets	500 W Xe lamp	Rhodamine B	3.5 h	72.5%	[47]
Octagonal nanosheets	300 W Xe lamp λ > 400 nm	Rhodamine B	2 h	61.7%	[48]
Microspheres	500 W halogen tungsten lamp λ > 420 nm	Methyl orange	3 h	15%	[49]
Microspheres	300 W Xe lamp λ > 400 nm	Methyl orange	180 min	51.1%	[50]
Microspheres	350 W Xe lamp λ > 420 nm	Carbamazepine	180 min	70%	[51]
Microspheres	300 W Xe lamp λ > 420 nm	Norfloxacin	120 min	57.8%	[52]
Microspheres	500 W iodine tungsten lamp white light of 380–830 nm	Bisphenol A	3 h	47.4%	[53]

## Data Availability

No additional data are available.
